# Stability of the HSV-2 *US-6* Gene in the del II, del III, *CP77*, and *I8R*-*G1L* Sites in Modified Vaccinia Virus Ankara After Serial Passage of Recombinant Vectors in Cells

**DOI:** 10.3390/vaccines8010137

**Published:** 2020-03-19

**Authors:** Vajini N. Atukorale, Jerry P. Weir, Clement A. Meseda

**Affiliations:** Division of Viral Products, Center for Biologics Evaluation and Research, U S Food & Drug Administration, Silver Spring, MD 20993, USA; v.atukorale@gmail.com (V.N.A.); jerry.weir@fda.hhs.gov (J.P.W.)

**Keywords:** modified vaccinia virus Ankara, vaccine stability, transgene, HSV-2

## Abstract

The modified vaccinia virus Ankara (MVA), a severely attenuated strain of vaccinia virus, is a promising vector platform for viral-vectored vaccine development because of its attributes of efficient transgene expression and safety profile, among others. Thus, transgene stability in MVA is important to assure immunogenicity and efficacy. The global GC content of the MVA genome is 33%, and GC-rich sequences containing runs of C or G nucleotides have been reported to be less stable with passage of MVA vectors in cells. The production of recombinant MVA vaccines requires a number of expansion steps in cell culture, depending on production scale. We assessed the effect of extensive passage of four recombinant MVA vectors on the stability of the GC-rich herpes simplex type 2 (HSV-2) *US6* gene encoding viral glycoprotein D (gD2) inserted at four different genomic sites, including the deletion (del) II and del III sites, the *CP77* gene locus (*MVA_009–MVA_013*) and the *I8R*-*G1L* intergenic region. Our data indicate that after 35 passages, there was a reduction in gD2 expression from del II, del III and *CP77* sites. Sequencing analysis implicated *US6* deletion and mutational events as responsible for the loss of gD2 expression. By contrast, 85.9% of recombinant plaques expressed gD2 from the *I8R*-*G1L* site, suggesting better accommodation of transgenes in this intergenic region. Thus, the *I8R*-*G1L* intergenic region may be more useful for transgene insertion for enhanced stability.

## 1. Introduction

MVA was derived by more than 570 passages of the chorioallantois vaccinia virus Ankara (CVA) in chicken embryo fibroblast (CEF) cells [1]. Genome analysis showed that serial passaging of CVA in cells resulted in deletions and mutations in several regions of the genome, particularly in the terminal regions. Genes located in the central region of the genome remained intact, as many are essential for virus replication. Six major deletions were mapped by restriction enzyme analyses and designated as del I through VI [2,3]. In addition, other less prominent deletions as well as several mutations in the genome have been described [4]. The sum total of the deletions from CVA (GenBank #: AM501482) that resulted in MVA (GenBank accession # AY603355.1 for Acambis 3000 MVA) was > 25 kilobase pairs, representing >13% of the entire virus genome [3], and includes genes encoding immune evasion and virulence factors. MVA is capable of infecting mammalian cells and expressing proteins but it is impaired in morphogenesis of infectious virions [5] due in part to the loss of the *C12L* gene encoding serine protease inhibitor-1 [6] and truncation of the *C16L*/*B22R* host-range gene [7]. MVA has been shown to be safe in humans, and a strain of MVA (MVA-BN) has been approved by regulatory authorities in Canada, the European Union [8], and the United States, as a prophylactic smallpox (and monkeypox in the USA) vaccine [9]. 

Orthopoxviruses, including vaccinia virus, canarypox virus and fowlpox virus, have been used as vectors for the expression of heterologous genes and are promising vaccine vectors for a variety of infectious diseases, as well as therapeutic vectors for the treatment of cancers. Generally, the capacity of vaccinia virus to accommodate large fragments of heterologous DNA and ability to induce high-level expression of transgenes make it an attractive viral vector in vaccine development. In addition, MVA has a high safety profile, including in immunocompromised individuals. Thus, recombinant MVA vectors expressing antigens of the human immunodeficiency virus [10], hepatitis C virus [11], malaria parasite [12], Ebola virus [13,14], and cytomegalovirus [15], among others, have been evaluated as candidate vaccines. MVA has also been used as a vector for the expression of tumor-associated antigens—notably, the 5T4 tumor-associated antigens [16] and MUC-1 [17]. 

The stability of a transgene in a virus vector is important to ensure immunogenicity and efficacy. Previously, we showed that the conserved *B5R* gene encoding an essential protein (B5) of the enveloped virion form of vaccinia virus was unmutated after serial passages of MVA in two non-permissive cell lines, Vero and MRC-5 cells [18]. The insertion of transgenes into MVA is usually accomplished by homologous recombination, in which a transgene of interest is inserted into the deletion regions [19,20,21,22], or at suitable intergenic regions [23]. The del II and del III regions have been used for the insertion of foreign genes [20,21,22,24]. While genes inserted in the deletion regions are efficiently expressed, the stability of heterologous genes in these deletion sites is often evaluated by 5–10 passages of recombinant MVA in cell culture, on average. The deletion of indigenous viral genes during passage of CVA in cells suggests that heterologous genes inserted in the deletion sites could be prone to deletion and/or mutations after repeated serial passages in culture, especially as MVA and MVA vectors are commonly produced in chick embryo fibroblast cells. Recombinant MVA vectors losing foreign gene expression have been reported [20,21,22,25]. From the time of preclinical development through manufacturing of clinical-grade product, the development and production of a viral vaccine or virus-vectored vaccine in cell culture require a series of passages, including virus seed and working seed expansions, as well as additional expansion steps during the manufacturing phase. Thus, it is especially important for a recombinant viral vaccine to be genetically stable in order to prevent undesirable changes in a transgene during vector expansion, such as could adversely impact vaccine characteristics, including immunogenicity and ultimately, efficacy. 

The stability of transgenes in MVA following prolonged serial passages in cells has not been thoroughly investigated. Transgenes with stretches of ≥ four G or C nucleotides have been reported to undergo mutational changes when inserted into MVA and passaged in cell culture [22]. MVA has a global GC content of 33%. In the current study, we constructed MVA vectors in which the herpes simplex virus type 2 (HSV-2) *US6* gene (with a 64.2% GC content; 23 C-runs of ≥ 4 Cs; and 4 G-runs of ≥ 4 Gs) was inserted at different loci in MVA, including del II and del III regions, as well as the *CP77* gene locus and the *I8R*-*G1L* intergenic region. The *US6* gene and reporter gene expression cassettes were inserted in forward and reverse orientations in *CP77* and *I8R-G1L* sites. These vectors were evaluated for stability after 35 serial passages in DF-1 cells. Our data indicate that *US6* was most stable in the *I8R*-*G1L* intergenic region, irrespective of orientation of the gene. Deletion of *US6* or a combination of mutational changes (base substitutions or insertions) with consequent abrogation of translational initiation and/or induction of frameshift mutations were detected in recombinant plaques that failed to express gD2 after 35 serial passages in cells.

## 2. Materials and Methods

### 2.1. Cells and Viruses

DF-1 cells, a spontaneously transformed chicken fibroblast cell line (UMNSAH/DF-1 (ATCC # CRL-12203)) and BHK-21 cells (ATCC # CCL-10) were obtained from the American Type Culture Collection (ATCC, Manassas, Virginia) and were grown in Dulbecco’s Minimal Essential Medium (DMEM) supplemented with 10% fetal bovine serum (FBS) and 0.05 mg/mL gentamicin. MVA, a gift from Dr. Linda Wyatt and Dr. Bernard Moss (NIAID/NIH), and recombinant MVA vectors were prepared and titered in DF-1 cells. 

### 2.2. Generation of Plasmid Shuttle Vectors

The shuttle vector pCAM17 was constructed to allow for recombination at the *CP77* locus in MVA. A 572 bp left-flanking sequence of *CP77* (coordinates 7091 to 7662 of ACAM3000; *GenBank* # AY603355.1) was amplified from MVA genomic DNA by PCR, using the primer pair CGGATCCCAAGTTAACTAATAAATAAAAAGT (CM-84) and CCGCTAGCATTGATAAATAAATTTTTTCTAGTGAT (CM-85). The amplicon was ligated with Zero Blunt TOPO vector (ThermoFisher, Carlsbad, CA, USA) and subcloned as a *Bam*HI-*Nhe*I fragment into pcDNA3.1(-) (ThermoFisher, Carlsbad, CA, USA) in which a *Bgl*II-*Nhe*I fragment encompassing the CMV promoter had been removed, to generate pcDNA3-LF. Similarly, a 600 bp right-flanking sequence of *CP77* (corresponding to coordinates 9607 to 10206 of ACAM3000) was amplified from MVA genomic DNA with the primer pair, GGATCCCTCATTTTCCAAATAGTCAAACATTTTGA (CM-86) and GCAAGCTTGTTAATAGAACAAAGTAAGAAAATAG (CM-87). The amplicon was cloned into Zero Blunt TOPO vector and subcloned as a *Bam*HI-*Hind*III fragment into pcDNA3-LF to generate pcDNA3-LF_RF. The primer pair, GCTAGCGCCTTTCATTTTGTTTTTTTCTATGCTA (CM-88) and GATATCAACCACTTTGTACAAGAAAGCTGAA (CM-89) was used to amplify a 2569 bp fragment from the destination vector pLW44*att*R [26]. The amplicon encompasses the following elements in the order: vaccinia p11 promoter, sequence of the enhanced green fluorescent protein (eGFP), vaccinia mH5 promoter, and reading frame B of the Gateway Conversion System (ThermoFisher Carlsbad, CA, USA). The amplicon was cloned into Zero Blunt TOPO vector and subcloned as a *Nhe*I-*Eco*RV fragment into pcDNA3-LF_RF to obtain the destination vector pCAM17. 

The shuttle vector pCAM18 was constructed to allow for recombination at the *I8R-G1L* intergenic site in MVA. The left- and right-flanking sequences of *CP77* in pCAM17 were substituted with the left- and right-flanking sequences at the *I8R*-*G1L* site, respectively. The *I8R*-*G1L* left flank was amplified from MVA genomic DNA with the primer pair, GGATCCCTTATATGAAGTCTATACAGCGAATAGA (CM-113) and GCTAGCTTAGTTATTATCTACAGGAACAAATATAGT (CM-114). The 500 bp amplicon (coordinates 57520 to 58019 in ACAM3000) was cloned into Zero Blunt TOPO vector, and subcloned as a *Bam*HI-*Nhe*I fragment into pcDNA3.1(-) that had been linearized with *Bgl*II and *Nhe*I to generate pcDNA3-18LF. Plasmid pCAM17 was digested with *Aat*II and *Nhe*I to excise the *CP77* left flank sequence. The *I8R*-*G1L* left flank was excised from pcDNA3-18LF as an *Aat*II-*Nhe*I fragment and ligated with the linearized pCAM17 to generate pCAM17-18LF. The primer pair, GATATCTCAAACTCTAATGACCACATCTTTTTTTAGA (CM-119) and AAGCTTATGATATACAGGCAATTATGGAATCT (CM-120b) was used to amplify the right flank of the *I8R*-*G1L* locus. The 500 bp amplicon (corresponding to coordinates 58023 to 58522 in ACAM3000) was cloned into Zero Blunt TOPO vector and subcloned as an *Eco*RV-*Hind*III fragment into pCAM17-18LF (in which the pCAM17 right flank sequence had been removed with *Eco*RV-*Hind*III) to generate pCAM18. 

Plasmids pCAM17R and pCAM18R were designed for the insertion of transgenes in leftward reading frames (Reverse orientation) at the *CP77* locus and *I8R*-*G1L* intergenic region, respectively. For pCAM17R, the primer pair, GCTAGCATCAACAAGTTTGTACAAAAAAGCTGA (CM-90; coordinates 1792 to 1818 of pLW44*att*R) and CGAGAAATAATCATAAATAAGCCCATCAACCACTTTGTACAAGAAAGCTGA (CM-79; containing coordinates 1768 to 1785 as well as the reverse complement of coordinates 3478 to 3501 in pLW44*att*R) was used to amplify the mH5 promoter, eGFP and p11 promoter cassette in reverse orientation from pLW44*att*R. The primer, GGGCTTATTTATGATTATTTCTCGCTT (CM-80; coordinates 1765 to 1791 in pLW44*att*R) was used with GATATCGCCTTTCATTTTGTTTTTTTCTATGCT (CM-91; coordinates 945 to 971 in pLW44*att*R) to amplify an 847 bp fragment that contained sequences of the p11 promoter, eGFP and mH5 promoter in the reverse orientation. The CM-90/CM-79 and CM-80/CM-91 PCR products were purified from gels and combined as templates for a second-step PCR with primers CM-90 and CM-91 to amplify a 2569 bp product that contains sequences in the order: cassette B Gateway Conversion System, and mH5/eGFP/p11 in the reverse orientation. The second-step PCR product was ligated with Zero Blunt TOPO vector and *ccdB* survival 2T1 competent *Escherichia coli* cells (ThermoFisher, Carlsbad, CA, USA) were transformed. Plasmid recovered from transformed cells was digested with *Nhe*I and *Eco*RV and the cassette B/ mH5/eGFP/p11 fragment was subcloned into pcDNA3-LF_RF to obtain pCAM17R. The pCAM18R shuttle vector was generated using the same strategy that was used for pCAM18, i.e., the *CP77* left- and right-flanking sequences in pCAM17R were substituted with the left- and right-flanking sequences at the *I8R*-*G1L* site to obtain pCAM18R.

### 2.3. Construction of Recombinant MVA Vectors Expressing gD2

The recombinant MVA-gD2 (MVAdel2-gD2) vector expressing HSV-2 (strain MS) gD2 from the MVA del II site has been described [24]. For insertion into the MVA del III site, *CP77*, and *I8R*-*G1L* domains, the HSV-2 (strain MS) *US6* gene encoding gD2 (GenBank accession # EU445527.1) was amplified from viral genomic DNA with the primers CACCATGGGGCGTTTGACCTCCGGCGT (CM-63; containing sequences corresponding to coordinates 141016 to 141038 of HSV-2 strain HG52, GenBank # Z86099.2) and CTAGTAAAACAATGGCTGGTGCGA (CM-62; corresponding to the reverse complement of coordinates 142174 to 142197). The amplicon was ligated with pENTR/D-TOPO vector (ThermoFisher, Carlsbad, CA, USA) to generate pENTR-*US6*. Isolates of pENTR-*US6* were sequenced to confirm nucleotide sequence authenticity. The plasmids pLW44-*US6*, pCAM17-*US6* and pCAM18-*US6* were generated by LR recombination between pENTR-*US6* and pLW44*att*R, pCAM17 and pCAM18, respectively, using the Gateway LR clonase enzyme mix (ThermoFisher, Carlsbad, CA, USA). pLW44-*US6*, pCAM17-*US6* and pCAM18-*US6* (1 µg each) were transfected into DF-1 cells that had been infected for 1 h with MVA at a multiplicity of infection (MOI) of 0.1, using X-tremeGENE HP DNA Transfection Reagent and protocol described by the manufacturer (Sigma-Aldrich, St. Louis, MO, USA). Recombinant MVA vectors—MVAdel3-gD2, MVA17-gD2 and MVA18-gD2—expressing gD2 from del III, *CP77* and *I8R*-*G1L* sites, respectively, were isolated by 3 to 5 rounds of plaque purification, expanded in DF-1 cells and partially purified on 36% sucrose cushions as previously described [27,28]. 

For the generation of recombinant MVA vectors with *US6* in the reverse orientation at *CP77* and *I8R*-*G1L* domains, the *US6* sequence was amplified from HSV-2 (strain MS) genomic DNA with the primer pair, CACCCTAGTAAAACAATGGCTGGTGCGA (CM-70; containing sequences corresponding to the reverse complement of coordinates 142174 to 142197 of HSV-2 strain HG52) and GGCATGGGGCGTTTGACCTCCGGCGT (CM-71; coordinates 141013 to 141038 of HSV-2 strain HG52). The PCR product was ligated with pENTR/D-TOPO vector to generate pENTR-US6R, and isolates were sequenced. The plasmids pCAM17-US6R and pCAM18-US6R were generated by LR recombination between pENTR-US6R and pCAM17R and pCAM18R, respectively, using the Gateway LR clonase enzyme mix. Recombinant MVA17-gD2rev and MVA18-gD2rev expressing gD2 from a leftward-reading *US6* sequence were generated and purified as described above. 

The growth curves of recombinant MVA vectors with *US6* inserted at the four different sites were similar to that of MVA (data not shown). 

### 2.4. Serial Passage of MVA-gD2 Vectors in Cells

Monolayers of DF-1 cells in 6-well tissue culture plates were infected with MVA-gD2 vectors, at an approximate MOI of 0.1. Following virus adsorption for 2 h, infection medium was aspirated, and 1 mL of fresh DMEM growth medium added to each well. Infected cells were incubated for 48 h, followed by three freeze–thaw cycles to release virions. The lysates were clarified by low-speed centrifugation at 1000 rpm for 10 min at 4 °C and stored at −80 °C as the inoculum for the next passage. This process was repeated 35 times for each recombinant MVA vector. MVAdel2-gD2 was also passaged 35 times in BHK-21 cells.

### 2.5. Western Blotting

In evaluating gD2 expression, DF-1 cells were infected with passage (P)1, P5, P10, P15, P20, P25, P30 and P35 of recombinant MVA vectors at a MOI of 1.0. Cells infected with MVA served as a control. Lysates of infected cells obtained 24 h after infection were tested for gD2 expression by Western blotting as previously described [24], but a 1:1000 dilution of rabbit anti-HSV-2 polyclonal antiserum (code B116, DAKO Corp., Waltham, MA, USA) was used as the primary antibody and an HRP-conjugated anti-rabbit antibody (ECL) at 1:5000 dilution was used as the secondary antibody. As a control, all lysates, along with a DF-1 cell control lysate, were tested for expression of the 14K vaccinia A27 protein by Western blotting, using a 1:250 dilution of a rabbit anti-A27 polyclonal antibody (a gift of Dr. Yong He, Division of Viral Products, CBER/FDA) as the primary antibody, and a HRP-conjugated anti-rabbit antibody (ECL) at 1:5000 dilution as the secondary antibody. The Supersignal West Dura substrate (Thermo Fisher, Rockford, IL, USA) was used as HRP substrate according to the manufacturer’s instruction. Images were captured under a LAS-3000 Luminescent Image Analyzer (Fujifilm Corp., Tokyo, Japan).

### 2.6. Immunostaining and Estimation of 50% Staining Endpoint (SE_50_)

Immunostaining of MVA plaques expressing gD2 was performed as previously described [27], with modifications. Confluent monolayers of DF-1 cells in a 6-well tissue culture plate were infected with recombinant MVA vectors at 100 PFU/well for 2 h. Infection medium was aspirated from wells and a 2 mL overlay of DMEM medium containing 5% FBS and 0.5% methyl-cellulose was added to each well. After a 48-h incubation, the overlay medium was aspirated, and cells were fixed with 1% paraformaldehyde for 10 min, and air dried. Rabbit anti-HSV-2 polyclonal antibody was diluted 1:1000 in Tris-buffered saline (TBS) containing 1% BSA (diluent), and 1 mL added to each well. After a 1.5-h incubation, cells were washed three times with TBS, and a 1:5000 dilution of an alkaline phosphatase-conjugated anti-rabbit antibody (Southern Biotech, Birmingham, AL, USA) was added to each well. After a 1-h incubation, cells were washed with TBS and 1 mL of Western blue-stabilized substrate for alkaline phosphatase was added to each well. After 5 to 10 min, the substrate was washed off with water, plates were air dried, and the number of stained and unstained plaques were counted in each well. The percentage of stained and unstained cells were calculated based on the total number of plaques (stained + unstained) in each well. For each recombinant MVA vector, the percentages of stained and unstained plaques at each passage were graphed using (GraphPad Software, Inc., San Diego, CA, USA). The number of passages at which 50% of plaques were stained for gD2 expression (defined as the 50% staining endpoint (SE_50_)) was extrapolated as the *x-axis* coordinate corresponding to the intercept of the stained/unstained curves. 

### 2.7. Isolation of Genomic DNA and PCR Assay

Monolayers of DF-1 cells were infected at a MOI of 1.0, with MVA or recombinant MVAdel2-gD2 plaque isolates. After a 24-h incubation, infected cells were harvested and collected by refrigerated (4 °C) centrifugation at 1000 rpm for 10 min. The cell pellet was re-suspended in 100 µL of PBS, and total genomic DNA was isolated from cells using the DNeasy blood and tissue kit, and protocol described by the manufacturer (Qiagen, Hilden, Germany). DNA was eluted from the column with 100 µL of ultrapure water, and the eluate was used as template in PCR assay, using the Phusion high-fidelity DNA polymerase, and protocol described by the manufacturer (New England Biolabs, Ipswich, MA, USA). For PCR assay, the primers CM-63 and CM-62 were used for the amplification of *US6*, and amplicons were ligated with PCR II Zero Blunt TOPO vector (ThermoFisher, Carlsbad, CA, USA), and sequenced with T7 and Sp6 promoter primers, and *US6*-specific internal primers. The sequencing data were aligned with published sequence data available in the database (GenBank accession # EU445527.1).

## 3. Results

### 3.1. Construction of Recombinant MVA Vectors Expressing HSV-2 gD2 from del II, del III, CP77 and I8R-G1L Intergenic Region

**Figure 1:**
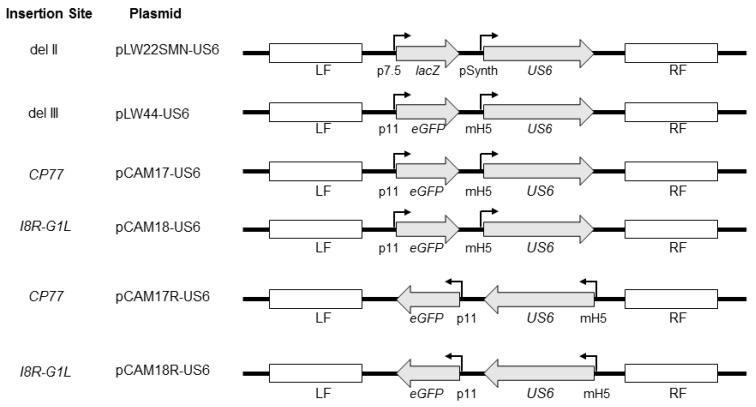
Schematic representation of plasmid shuttle vectors used in the construction of recombinant modified vaccinia virus Ankara (MVA) vectors. The shuttle vector pLW22SMN-gD2 which contains the HSV-2 *US6* gene under a synthetic vaccinia early/late promoter (pSynth), along with a *lacZ* reporter gene under the vaccinia p7.5 promoter, was previously described [24]. The *lacZ* and *US6* expression cassettes are flanked by MVA sequences flanking the del II site in MVA. pLW44-gD2 contains the sequence of enhanced green fluorescent protein (eGFP) under the vaccinia P11 promoter and the *US6* gene under the modified vaccinia mH5 promoter. The eGFP and *US6* expression cassettes are flanked by MVA sequences flanking the deletion III site in MVA. pCAM17-US6 and pCAM17R-US6 contain an eGFP expression and a *US6* expression cassettes identical to pLW44-US6 but the expression cassettes are flanked by MVA sequences flanking the *CP77* gene locus. The eGFP and *US6* expression cassettes in pCAM17R-US6 are in leftward-reading orientation. The expression cassettes in pCAM18-US6 and pCAM18R-US6 are identical to those in pCAM17-US6 and pCAM17R-US6, respectively, except that they are flanked by flanking sequences of the vaccinia *I8R*-*G1L* intergenic region. The direction of *US6* and reporter genes is shown with arrows. Flanking sequences for homologous recombination at the site of gene insertion are shown as LF (left flank) and RF (right flank).

In order to evaluate the stability of the HSV-2 *US6* gene in MVA, the gene was inserted into four regions of the MVA genome, including the del II and del III regions, the *CP77* locus, and the *I8R*-*G1L* intergenic region, by homologous recombination of MVA with plasmid shuttle vectors (Figure 1). 

The del II site carries an approximately 3.5 kilobase pair (kbp) deletion located toward the left terminal of the MVA genome, and the del III site carries an approximately 4.7 kbp deletion located toward the right terminal of the genome. These del regions were selected in this study primarily because of the availability of plasmid shuttle vectors for homologous recombination of transgenes into these sites, and the del III site is frequently used as a gene insertion site for candidate MVA-vectored vaccines in preclinical studies. Previously, we described the construction of the MVA vector expressing gD2 from the del II site [24]. For the insertion of the *US6* gene in del III, we used the pLW44*att*R shuttle vector. The shuttle vectors pCAM17/pCAM17R and pCAM18/pCAM18R were constructed and used for the insertion of *US6* into the *CP77* locus and *I8R*-*G1L* intergenic region, respectively. Purified recombinant MVA vectors (Table 1) were tested by PCR for the absence of MVA sequences at the sites of homologous recombination and expression of gD2 protein was confirmed by Western blotting (data not shown). 

### 3.2. Stability of US6 in the del II and del III Regions

**Figure 2:**
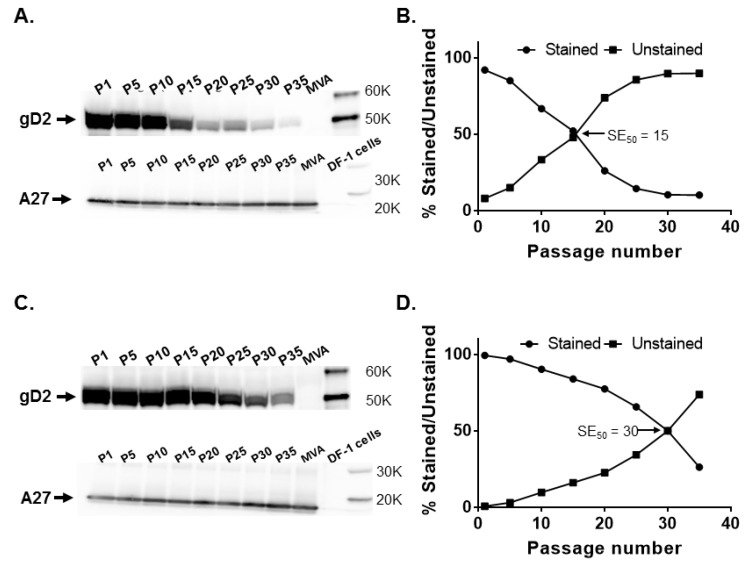
Stability of the HSV-2 *US6* gene in MVA deletion (del) II and del III sites. Recombinant MVAdel2-gD2 and MVAdel3-gD2 were passaged 35 times in DF-1 cells and tested for glycoprotein D2 (gD2) expression by Western blotting and immunostaining at passages (P) 1, 5, 10, 15, 20, 25, 30, and 35, along with MVA-infected cell controls. (**A**) gD2 and vaccinia A27 protein expression by MVAdel2-gD2 evaluated by Western blotting and (**B**) by immunostaining. (**C**) gD2 and vaccinia A27 protein expression by MVAdel3-gD2 assessed by Western blotting and (**D**) by immunostaining. The SE_50_ is the estimated number of passages of recombinant MVA at which 50% of plaques stained positive for gD2 expression with a rabbit anti-HSV-2 polyclonal antibody. SE_50_ was determined as the *x*-axis coordinate corresponding to a *y*-axis coordinate of 50%.

Recombinant MVAdel2-gD2 and MVAdel3-gD2 vectors were analyzed for gD2 expression by Western blotting and by immunostaining at intervals of five passages. The former assay allows for a qualitative determination of gD2 expression, while the latter allows for a quantitative determination of the proportion of recombinant plaques expressing gD2. In Western blotting analysis, decreasing levels of gD2 expression from both del II and del III sites were detected with increasing in vitro passage but a similar level of expression of the 14K viral A27 protein was obtained at the different passage levels (Figure 2A,C). However, the decrease in gD2 expression appeared to be more pronounced with increasing passage of MVAdel2-gD2. 

These observations were confirmed in immunostaining, where the percentage of plaques expressing gD2 had decreased to 52.1% at P15 and was 14.4% and 10.2% by P25 and P35, respectively. The SE_50_ for MVAdel2-gD2 was determined to be 15.4 (Figure 2B). By comparison, 83.9% of MVAdel3-gD2 plaques stained for gD2 expression at P15, decreasing to 65.7% by P25, with the SE_50_ determined to be 30 (Figure 2D). This set of data indicates that gD2 expression from both del II and del III sites decreased with an increase in passage, but *US6* in the del III site appears to be relatively more stable than in the del II site. 

### 3.3. Stability of US6 at the CP77 Locus and I8R-G1L Intergenic Region

**Figure 3:**
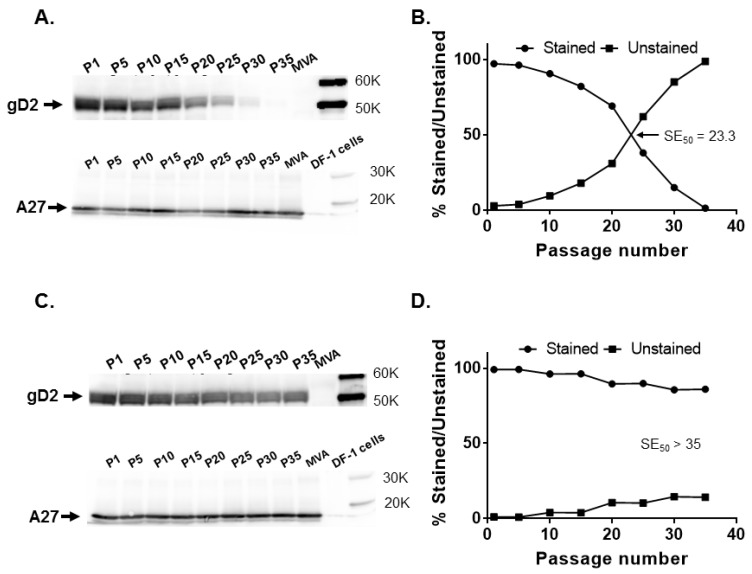
Stability of the HSV-2 *US6* gene in rightward orientation at the *CP77* locus and *I8R*-*G1L* intergenic site. Recombinant MVA17-gD2 and MVA18-gD2 were passaged 35 times in DF-1 cells and tested for gD2 expression by Western blotting and immunostaining at passages (P) 1, 5, 10, 15, 20, 25, 30, and 35, along with MVA-infected cell controls. (**A**) gD2 and vaccinia A27 protein expression by MVA17-gD2 evaluated by Western blotting and (**B**) by immunostaining. (**C**) gD2 and vaccinia A27 protein expression by MVA18-gD2 assessed by Western blotting and (**D**) by immunostaining. The SE_50_ is the estimated number of passages of recombinant MVA at which 50% of plaques stained positive for gD2 expression with a rabbit anti-HSV-2 polyclonal antibody. SE_50_ was determined as the *x*-axis coordinate corresponding to a *y*-axis coordinate of 50%.

When different passages of MVA17-gD2 were tested for gD2 expression, protein expression decreased with an increase in virus passage despite similar levels of expression of A27 (Figure 3A), with 82.1% of plaques staining positive for gD2 at P15 and dropping to 38% by P25. The SE_50_ for MVA17-gD2 was determined to be 23.3 (Figure 3B). 

Upon serial passage of MVA18-gD2, a decrease in gD2 expression level was not readily discernible by Western blotting and the levels of A27 expression were also similar (Figure 3C). In immunostaining, 96.2% of MVA18-gD2 plaques expressed gD2 at P15 and 85.9% expressed gD2 at P35 (Figure 3D). Thus, in agreement with the predicted stability of transgenes inserted in the conserved orthopoxvirus genome region harboring genes encoding indispensable gene products, the HSV-2 *US6* gene in the *I8R*-*G1L* intergenic region remained stable after extensive passage of MVA18-gD2 in cells.

### 3.4. Effect of Gene Orientation on Transgene Stability in MVA

**Figure 4:**
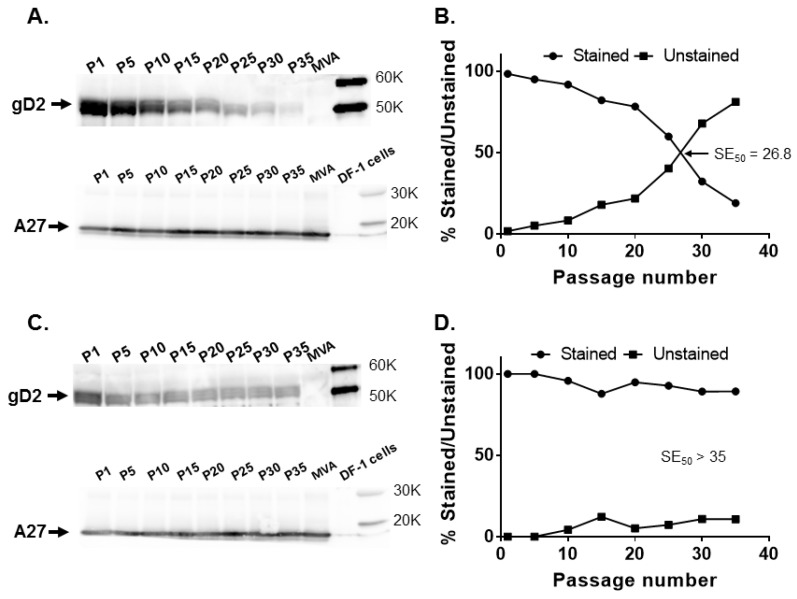
Stability of the HSV-2 *US6* gene in leftward orientation at the *CP77* locus and *I8R*-*G1L* intergenic site. Recombinant MVA17-gD2rev and MVA18-gD2rev were passaged 35 times in DF-1 cells and tested for gD2 expression by Western blotting and immunostaining at passages (P) 1, 5, 10, 15, 20, 25, 30, and 35, along with MVA-infected cell controls. (**A**) gD2 and A27 expression by MVA17-gD2rev evaluated by Western blotting and (**B**) by immunostaining. (**C**) gD2 and A27 expression by MVA18-gD2rev assessed by Western blotting and (**D**) by immunostaining. The SE_50_ is the estimated number of passages of recombinant MVA at which 50% of plaques stained positive for gD2 expression with a rabbit anti-HSV-2 polyclonal antibody. SE_50_ was determined as the *x*-axis coordinate corresponding to a *y*-axis coordinate of 50%.

The organization of large DNA viral genomes such as herpesviruses and poxviruses have ORFs that read in either a rightward or a leftward direction. The majority of the ORFs around the *CP77* locus are in the leftward-reading orientation. In the *I8R*-*G1L* region, the two adjacent genes (*I8R* and *G1L*) are in opposite transcriptional orientations, with *I8R* being rightward-reading and *G1L* being leftward-reading. To assess whether the orientation of *US6* has any impact on its stability in MVA, the *US6* gene was inserted in leftward (reverse) orientation at the *CP77* locus and *I8R*-*G1L* intergenic region to generate MVA17-gD2rev and MVA18-gD2rev, respectively. A gradual decrease in gD2 expression was observed when MVA17-gD2rev was passaged in cells but without a decrease in A27 expression (Figure 4A). 

At P15, 82.1% of MVA17-gD2rev plaques expressed gD2, while 59.8% expressed gD2 at P25. An SE_50_ of 26.8 was determined for MVA17-gD2rev (Figure 4B)—slightly higher than the 23.3 passages obtained for MVA17-gD2. By contrast, gD2 as well as A27 expression were consistent for MVA18-gD2rev (Figure 4C) and 89.2% of plaques expressed gD2 at P35 (Figure 4D). This set of data suggests that, rather than orientation, the genomic location of a transgene in MVA may be more important in enhancing its stability, and insertion in the *I8R*-*G1L* intergenic region enhanced stability of *US6*.

### 3.5. Mutations and Gene Deletion Account for US6 Instability in the del II Site in MVA

As described above, more than 50% of recombinant MVA vectors no longer expressed gD2 after 15 to 30 passages when *US6* was inserted in the del II, del III, or *CP77* locus in MVA. In order to characterize the increasing number of non-gD2 expressing plaques, the P35 of MVAdel2-gD2 originally passaged in BHK-21 cells was titrated on DF-1 cells. This passage was selected because preliminary immunostaining assay indicated that it was almost uniformly non-gD2 staining, thereby increasing our chances of picking non-gD2 staining plaques. Ten (10) plaques were randomly selected, isolated and expanded in cells. In Western blotting, none of the 10 plaque isolates expressed gD2 (not shown). Genomic DNA was extracted from each isolate and used as a PCR template to amplify the *US6* gene. US6 was amplifiable from 4 of the 10 plaque isolates. To verify the gD2 expression status of the isolates, two US6 PCR^positive^ and two US6 PCR^negative^ isolates were titered on DF-1 cells, and infected cells were immunostained for gD2 expression. None of the isolates had detectable plaques stained with HSV-2 antibody. This set of data suggests that the *US6* gene may have been excised from the MVA vector (PCR^negative^ isolates) or mutated (PCR^positive^ isolates) during in vitro passage in cells. 

In order to further evaluate the lack of gD2 expression, the four *US6* sequences amplified by PCR above were ligated with Zero Blunt TOPO plasmid vector and two plasmid DNA isolates from each were sequenced using T7 promoter and Sp6 promoter primers, along with *US6*-specific primers. Analyses of the sequence data revealed that each PCR^positive^ isolate contained at least two combinations of five different mutations, including an A to T substitution at position 1 that altered the **A**UG (Met) translational initiation codon to **UU**G (Leu) (Table 2). With the exception of one isolate, the ATG to TTG mutation, along with a **C**GC to **T**GC (Arg to Cys) substitution at position 1032, was present in all isolates (Table 2). 

The other three types of mutation detected included a C deletion after a series of seven C nucleotide run at coordinates 165 to 171 of the *US6* sequence (present in all isolates); a C insertion after a series of six C nucleotide run at coordinates 305 to 310 (found in three isolates); and a C insertion after a series of six C nucleotide run at coordinates 945 to 950 (present in two isolates). Each pair of DNA isolate originating from the same non-gD2 expressing plaque had identical sets of mutations, except for sequences from clone 1, where isolate 1-1 did not contain the ATG to TTG mutation at position 1 (Table 2). Two of the eight isolates contained all four mutations. This set of data suggests that gene deletion and mutational events were primarily responsible for the instability of *US6* in the del II site in MVA. 

## 4. Discussion

Interest in the use of MVA as a vaccine vector has soared in the past decade. This interest is due in part to its well-documented safety profile, ability for high-level expression of transgenes and capacity to induce robust humoral and cellular immune responses. The stability of a transgene in a viral vector is critical for its successful use as a platform for vaccine development. As MVA and MVA-vectored vaccines are produced in cell culture, we evaluated the stability of a transgene in MVA after several serial passages in cell culture. For this purpose, the HSV-2 *US6* gene with a GC content of > 64% was selected because of its relatively high GC content compared to the ~ 33% global GC content of MVA. The high GC content of *US6* is a potential recipe for instability in the low-GC background of MVA, making it an ideal transgene for this study. 

The *US6* gene was inserted at different sites in MVA (Figure 1) and assessed for stability by serial passage of recombinant MVA vectors (MVA-gD2) in cell culture. Gradual decreases in gD2 expression were observed after prolonged serial passage of the vectors, irrespective of the site of insertion of *US6*. Overall, MVAdel2-gD2, with *US6* inserted in the MVA del II site, appeared to be more prone to instability as almost half of plaques failed to express gD2 by P15 (Figure 2). By comparison, MVAdel3-gD2 had a higher SE_50_ value (Figure 2). This set of data suggests that of the two MVA deletion sites evaluated, *US6* was more stable in the del III site. 

*CP77* is a gene that encodes a 77 kDa host-range protein that was described in cowpox virus (CPXV; corresponds to open reading frame (ORF) *CPXV025* in CPXV strain Brighton Red (*GenBank* # AF482758.2) and ORF *CVA017–CVA021* in CVA) and responsible in part for the permissiveness of productive CPXV replication in CHO cells [29]. In vaccinia virus strains, including MVA and CVA, *CP77* is present but fragmented into 4-5 pieces and non-functional. Absence of the *CP77* gene product accounts in part for the lack of permissiveness of CHO cells for vaccinia virus replication [29]. In a previous study [30], we described the insertion of the firefly luciferase gene at the *CP77* locus in the IHD-J and Western Reserve (WR) strains of vaccinia virus. Passage of IHDJ-luc but not WR-luc in cells resulted in a noticeable reduction in luciferase expression. A loss of gD2 expression was observed with increasing passage of MVA vectors with *US6* at the *CP77* locus (Figure 3 and Figure 4). 

The vaccinia *I8R* and *GIL* genes encode an RNA/DNA helicase and an insulin metalloproteinase-like protein, respectively. Both *I8R* and *GIL* are located in the well-conserved region of vaccinia virus genome, separated by three adenine nucleotides, and *G1L* is essential for vaccinia virus replication [31,32]. The *I8R*-*GIL* intergenic locus has previously been used for the insertion of heterologous genes [22,33]. It has been reported that transgenes inserted in the conserved central region housing essential chordopoxviruses genes are maintained. For example, Wyatt et al. [22] observed that >99% recombinant MVA plaques in which the HIV (clade UGD) *env* gene was inserted in the intergenic site between the *I8R* and *G1L* genes stained for gp41expression after five passages in chicken embryo fibroblast cells. We evaluated the stability of *US6* inserted in the *I8R* and *G1L* intergenic region by a more extensive serial passage of MVA18-gD2. Our data (Figure 3) indicate continuous stability of *US6* in the *I8R*-*G1L* intergenic site. Insertion of *US6* in either rightward or leftward orientation at the *CP77* locus or *I8R*-*G1L* intergenic region had no significant effect on gD2 expression (Figure 4), suggesting that topological orientation has little or no effect on transgene stability in MVA. 

In general, MVA gene expression was not affected by the passage of recombinant MVA-gd2 vectors in cells, as evidenced by the similar levels of vaccinia A27 protein detected irrespective of the number of passages of MVA vectors in cells. 

Further gene expression and sequencing analyses indicated that gene deletion and mutational events contributed to the loss of gD2 expression after passage of MVA vectors in cells (Table 2), including mutations with resultant frameshifts. A point mutation at nucleotide position 1 resulted in a change in the translational initiation codon to UUG. Although a UUG codon is functional as an alternative initiation codon in some prokaryotes (e.g., for the *lacA* gene encoding thiogalactoside acetyltransferase in *Escherichia coli* (GenBank accession # J01636.1)), it is not a functional translation initiation codon in eukaryotic cells. Thus, recombinant MVA-gD2 plaques carrying this mutation will not be able to initiate the translation of *US6* messenger RNA into gD2, accounting in part for the absence of gD2 expression. Wyatt et al. [22] reported base insertion or deletion after four or more C- or G-runs as the major form of mutation in recombinant MVA vectors containing HIV genes. The HSV-2 *US6* gene used in this study carries a total of 23 stretches of C nucleotide of ≥ 4 (comprising ten 4 C-runs, seven 5 C-runs, five 6 C-runs, and a 7 C-run). Four stretches of G nucleotide (comprising three 4 G-runs and a 5 G-run) are also present in *US6*. However, the base insertions or deletions detected in our study occurred only after six or seven C-runs. Of note, the only stretch of seven C nucleotides is adjacent to a stretch of four G nucleotides, and all mutants contained a C-insertion after the stretch of seven C nucleotides, suggesting that DNA regions with higher numbers of nucleotides in a stretch of Cs are more prone to base insertion or deletion. Thus, important consideration should be given to the nucleotide sequence of a transgene that is to be recombined into MVA. The introduction of silent codon changes to disrupt G- or C-runs [22] or chemical synthesis of genes can be used to prevent the occurrence of G- and C-runs in transgenes prior to recombination with MVA.

## 5. Conclusions

Taken together, our data underscore the need for a thorough assessment of a combination of various factors, including insertion site and the sequence of a transgene, in the design and construction of recombinant MVA-vectored vaccines. Such considerations are paramount for transgene stability, as they could impact the effectiveness of MVA-vectored vaccines. In addition, extensive passage of recombinant MVA vectors is not recommended during the production of MVA-vectored vaccines.

## Figures and Tables

**Table 1:** Recombinant modified vaccinia virus Ankara (MVA) constructs expressing HSV-2 glycoprotein D2 (gD2).

**Name**	**US6 Insertion Site**	**Plasmid Shuttle Vector**	**Vaccinia Promoter**	**US6 Gene Orientation**	**Reporter Gene/Orientation**	**Reference**
**MVAdel2-gD2**	del II	pLW22-SMN	Synthetic early/late	RRF	*lacZ*/RRF	[24]
**MVAdel3-gD2**	del III	pLW44attR *	mH5	RRF	GFP/RRF	[26]
**MVA17-gD2**	*CP77*	pCAM17 *	mH5	RRF	GFP/RRF	-
**MVA17-gD2rev**	*CP77*	pCAM17R *	mH5	LRF	GFP/LRF	-
**MVA18-gD2**	*I8R*-*G1L*	pCAM18 *	mH5	RRF	GFP/RRF	-
**MVA18-gD2rev**	*I8R*-*G1L*	pCAM18R *	mH5	LRF	GFP/LRF	-

* Destination vector contains Reading Frame B of the Gateway Vector Conversion System (ThermoFisher, Carlsbad, CA, USA); mH5, modified vaccinia H5 promoter [21]; RRF, Rightward-Reading Frame; LRF, Leftward-Reading Frame.

**Table 2:** Mutational changes in *US6* following serial passage of MVA-gD2 in cells.

**Isolate**	**Mutational Changes**					
	**ATG to TTG at Position 1**	**C deletion at Coord. 165–171 (Run of 7 Cs)**	**C insertion at Coord. 305–310 (Run of 6 Cs)**	**C Insertion at Coord. 945–950 (Run of 6 Cs)**	**CGC to TGC at Position 1032**	**Potential Effect of Mutation**
**1-1**	-	+	-	-	+	^1^ FM
**1-2**	+	+	-	-	+	^2^ NTI, FM
**2-1**	+	+	-	-	+	NTI, FM
**2-2**	+	+	-	-	+	NTI, FM
**3-1**	+	+	-	-	+	NTI, FM
**3-2**	+	+	-	-	+	NTI, FM
**4-1**	+	+	+	+	+	NTI, FM
**4-2**	+	+	+	+	+	NTI, FM

^1^ Frameshift mutation; ^2^ no translational initiation.
